# Predictive, preventive, personalised and participatory periodontology: State of knowledge and future perspectives

**DOI:** 10.1186/1878-5085-5-S1-A18

**Published:** 2014-02-11

**Authors:** Carlo Cafiero

**Affiliations:** 1 University of Naples Federico II, Naples, Italy

## 

The Springer Textbook in preparation is focused on periodontitis (a very frequent oral disease in population worldwide) and its multiple negative impacts on quality of life. A frequent sequaela of periodontitis is edentulism that carry with it a high social cost in terms of time and money. Moreover scientific evidence suggests that periodontal disease may play an aetiologic role in the pathogenesis of several condition of illness such as cardiovascular disease, preterm birth and diabetes. In consequence periodontitis should be considered not more as a mere infection of the mouth but as a disease that can put at risk general health. At this regard the book intends to provide the current knowledge on predictive, preventive and personalised periodontology with particular reference to high tech diagnostic tools such as lab-on-chip for chair side diagnostic tests. Finally, personalised therapy with tailored treatment respect to the particular medical reality of the specific stratified patient will be proposed in the text.

